# Trigger point injection for post-mastectomy pain: a simple intervention with high rate of long-term relief

**DOI:** 10.1038/s41523-021-00321-w

**Published:** 2021-09-17

**Authors:** Amal L. Khoury, Holly Keane, Flora Varghese, Ava Hosseini, Rita Mukhtar, Suzanne E. Eder, Philip R. Weinstein, Laura J. Esserman

**Affiliations:** 1 Department of Surgery, University of California San Francisco, East Bay - Highland Hospital, Oakland, CA USA; 2 Peter MacCallum Cancer Centre, Melbourne, VIC Australia; 3 General Surgery, Adventist Health and Rideout, Yuba City, CA USA; 4 Department of Surgery, University of California-San Diego, San Diego, CA USA; 5 Department of Surgery, University of California, San Francisco, San Francisco, CA USA

**Keywords:** Surgical oncology, Breast cancer

## Abstract

Post-mastectomy pain syndrome (PMPS) is a common and often debilitating condition. The syndrome is defined by chest wall pain unresponsive to standard pain medications and the presence of exquisite point tenderness along the inframammary fold at the site of the T4 and T5 cutaneous intercostal nerve branches as they exit from the chest wall. Pressure at the site triggers and reproduces the patient’s spontaneous or motion-evoked pain. The likely pathogenesis is neuroma formation after injury to the T4 and T5 intercostal nerves during breast surgery. We assessed the rate of long-term resolution of post-mastectomy pain after trigger point injections (2 mL of 1:1 mixture of 0.5% bupivacaine and 4 mg/mL dexamethasone) to relieve neuropathic pain in a prospective single-arm cohort study. Fifty-two women (aged 31–92) who underwent partial mastectomy with reduction mammoplasty or mastectomy with or without reconstruction, and who presented with PMPS were enrolled at the University of California San Francisco Breast Care Center from August 2010 through April 2018. The primary outcome was a long-term resolution of pain, defined as significant or complete relief of pain for greater than 3 months. A total of 91 trigger points were treated with mean follow-up 43.9 months with a 91.2% (83/91) success rate. Among those with a long-term resolution of pain, 60 trigger points (72.3%) required a single injection to achieve long-lasting relief. Perineural infiltration with bupivacaine and dexamethasone is a safe, simple, and effective treatment for PMPS presenting as trigger point pain along the inframammary fold.

## Introduction

Post-mastectomy pain syndrome (PMPS) is reported to affect 25–60% of women after breast cancer surgery^1^. Post-surgical pain can occur after any type of breast surgery including partial mastectomy, breast reduction, or axillary surgery^2^. Pain following surgical procedures such as mastectomy and reduction mammoplasty, where tissue is disrupted in the area of the T4 and T5 intercostal nerve sensory branches as they emerge from the chest wall to supply the breast, may have an etiology that is easily treatable.

Despite its prevalence, PMPS is infrequently recognized by surgeons or addressed clinically. Even when it is diagnosed, the majority of patients do not receive effective treatment^3^. Those who are accurately diagnosed and treated with standard medications such as opioid analgesics, non-steroidal anti-inflammatory drugs (NSAIDs), anticonvulsants (gabapentin), benzodiazepines, and antidepressants (SSRIs), often report disappointing results^4^. Therefore, many patients with PMPS are left with debilitating pain and report decreased quality of life^5^.

Acute injury to the sensory branches of cutaneous nerves during surgery can lead to neuroma formation and subsequent growth of an axonal cell membrane that develops pacemaker-like activity resulting in continuous firing. Clinically, this manifests as a neuropathic pain syndrome associated with persistent and often debilitating pain^6^. It can be persistent, stimulus-independent pain, and be triggered or further aggravated mechanically by pressure at the site of the neuroma.

This type of injury can be diagnosed by identifying a “trigger point,” which is defined as a reproducible focal or radiating pain activated by touching the site. Pain treatment specialists have found that perineural infiltration of dexamethasone in combination with local anesthetic at the site of the presumed neuroma may be effective in alleviating pain induced by touching these trigger points^7^. Infiltration with a mixture of bupivacaine and dexamethasone can be effective in treating neuromas^8,9^. Local anesthetic alone provides only short-term relief, but maybe diagnostic. Corticosteroids can inhibit ongoing discharge in chronic neuromas by suppressing the pacemaker activity which results in either rapid or delayed onset of pain relief^8^.

We hypothesized that one of the causes of PMPS is likely a neuropathic pain condition resulting from damage to the T4 and T5 peripheral nerves of the chest wall during surgery, resulting in hypersensitivity^10^ and neuroma formation, a major cause of chronic neuropathic pain post-mastectomy^7^. This mechanism is seen after other surgical procedures, such as inguinal nerve injury from surgical hernia repair surgeries^9^. Trigger point injections (TPI) have been used successfully for chronic pain after hernia repair.

The same principle was utilized to treat the neuropathic pain of PMPS. We hypothesized that the offending cause was a stretch, coagulation, or transection injury to the T4 and T5 cutaneous intercostal nerve branches. These branches are small in caliber and difficult to visualize during surgery. They are often accompanied by a vessel. Transection of the vessel and subsequent cauterization adjacent to where the T4 and T5 nerves cross the chest wall and enter the breast is thus a likely genesis of injury to the nerves. The procedures where this is most likely to occur are mastectomy and reduction mammoplasty, a common technique used to improve cosmesis or remove larger segments of the breast. We surmised that patients whose pain is a result of nerve injury might have trigger point pain at the site of the neuroma, in the specific regions where the T4 and T5 nerves cross the chest wall. We hypothesized that a clinical exam might then identify the location of the neuroma and a local injection at that site could relieve the pain.

Utilizing this principle, we initiated a quality improvement project to treat PMPS. This led to remarkable, long-lasting relief of the first few patients^11^, and we, therefore, continued to treat patients with clinical symptoms suggestive of a neuroma. We report on long-term pain relief after trigger point injections for a consecutive series of women who presented with PMPS characterized by a trigger point at the Carol Franc Buck Breast Care Center at the University of California San Francisco (UCSF).

## Results

### Study design

Ninety-one trigger points on 52 patients with PMPS were identified at the UCSF Breast Care Center from August 2010 through April 2018.

Patient age ranged from 31 to 92 years (mean 53 years). The mean number of surgeries prior to injection was 2.2 ranging from 1 to 8. In this cohort, we found that the most frequent surgical procedure that preceded the development of a neuroma was mastectomy, followed by reduction mammoplasty with or without concurrent partial mastectomy. The time from the onset of neuropathic pain to the first trigger point injection varied from as early as 1 week postoperatively to 144 months (mean 22.5 months). Descriptive patient characteristics are found in Table 1. Data regarding the type of surgical procedures, any associated surgical complications, and radiation therapy are listed in Table 2.

**Table 1:** Descriptive patient characteristics.

Patient characteristics	Overall (*n* = 52) mean or %
Age at 1st injection (range in years)	52.2 (31–92)
Trigger points per patient (range)	1.8 (1–4)
Bilateral trigger points	19.2% (10)
Duration of pain prior to TPI (months, range)	23.4 (0.25–144)
US-guided injection	3.9% (2)

**Table 2:** Local management of breast cancer and complications.

Local management of breast cancer	Overall (*n* = 52) mean or %
Type of breast surgery	
% Lumpectomy	5.8
% Reduction ± lumpectomy	23.1
% Mastectomy (± recon)	71.1
Type of axillary surgery	
% None	19.2
% Sentinel node biopsy	51.9
% Axillary node dissection	28.9
# Breast operations prior to injection	2.3 (1–8)
Adjuvant radiotherapy %	38.5
Surgical complications	
% Yes (*n* = 16)	30.8
% No (*n* = 36)	69.2
Surgical complication severity	
% None	69.2
% Minor	19.2
% Major	11.5

*n* number of patients.

**Fig. 1:**
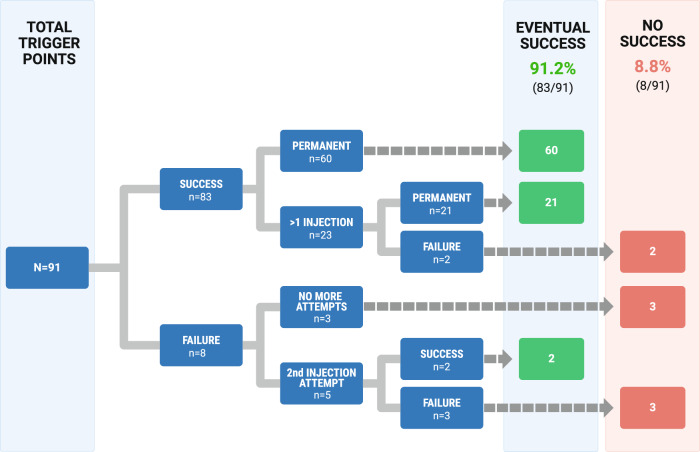
Overall outcomes for trigger points.

Two of the patients were lost to follow-up, leaving 91 trigger points in this analysis with long-term follow-up data (≥3 months). Long-term pain relief was achieved with 83 trigger points (91.2%). The majority of trigger points, 60 of 91 total trigger points, had resolution of pain following a single injection. A repeat injection was necessary to achieve eventual success for 23 trigger points (25.3%). There were 8 trigger point injections (9.0%) that failed to provide relief with the first attempt. However, two of these were re-treated and achieved long-term resolution of pain with a second injection (Fig. 1).

Patients with relief were evaluated to determine which characteristics may increase the likelihood of relief with TPI. These features included a number of surgeries prior to initial injection, history of complications resulting from their prior breast operation(s), period of time with pain (in months), age at first injection, and the number of times each trigger point was injected. The higher number of surgeries prior to injection, any surgical complications, and major surgical complications were all statistically significant on unadjusted bivariate analysis. Bivariate analysis characteristics and primary outcome are listed in Table 3. On multivariate analysis, only the severity of surgical complications remained statistically significant for the inability to achieve pain relief. TPI were well tolerated by all patients. There were no observed or reported complications associated with this treatment. No patient reported aggravation of pain by the treatment.

**Table 3:** Unadjusted comparisons between sample characteristics and primary outcome.

Variables	Means or % relief	*p*-value
Age at 1st injection	53.6	0.08
# Operations prior to injection	2.14/3	0.04
Period of time with pain in months	22.0	0.39
# Injections per TP	1.29	0.06
Type of breast surgery		
% Lumpectomy	100	0.23
% Reduction ± lumpectomy	100	
% Mastectomy (± recon)	86.9	
% Other	100	
Type of axillary surgery		
% None	87.1	0.54
% Sentinel node biopsy	91.9	
% Axillary node dissection	95.7	
Adjuvant radiotherapy %	96.6	0.22
Surgical complications		
% Yes (*n* = 31)	80.7	0.01
% No (*n* = 60)	96.7	
Surgical complication severity		
% Minor	88.5	0.003
% Major	66.7	

## Discussion

**Fig. 2:**
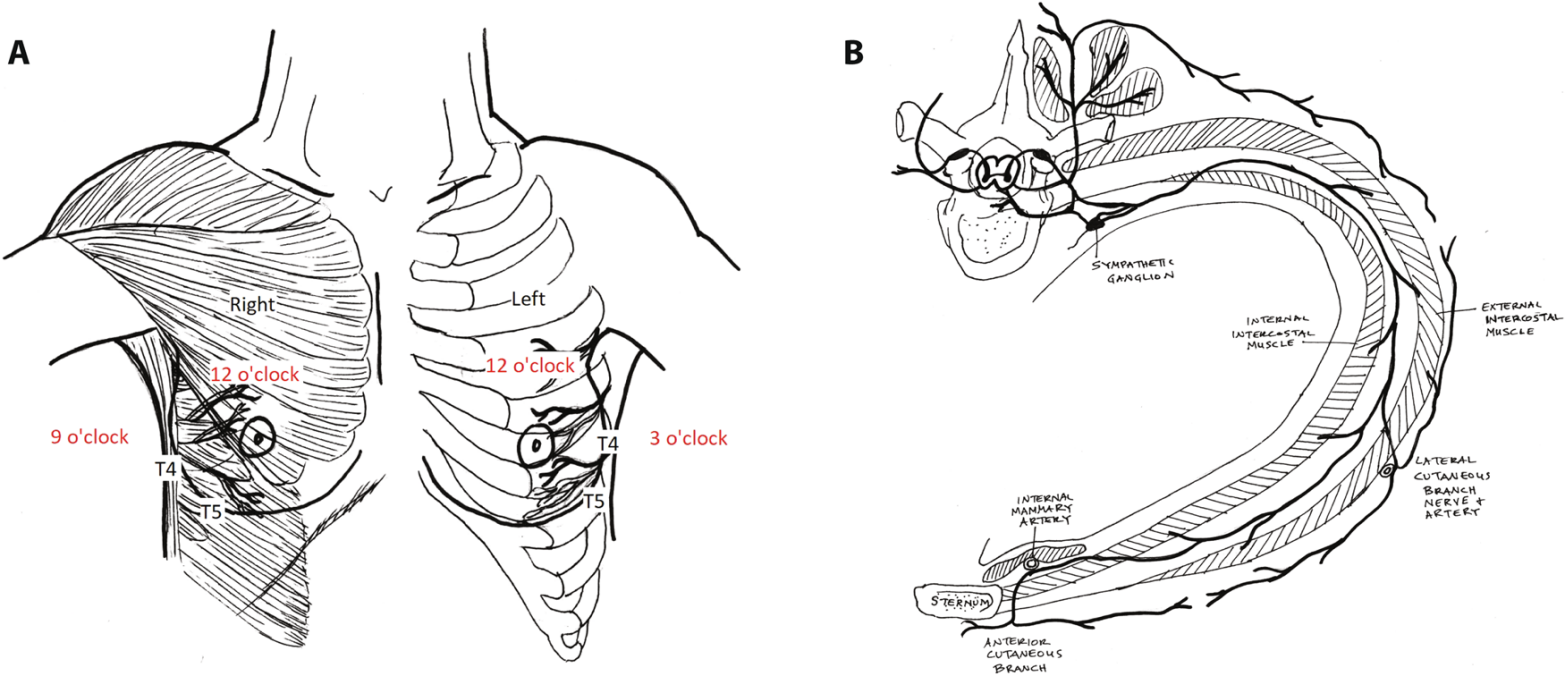
Anatomy of trigger point injections for post-mastectomy pain syndrome. **a** Trigger point locations. The patient is placed in the supine position and the breast (native or reconstructed) is lifted or retracted. The area just above the inframammary fold is examined, by palpating the rib, then firm pressure is maintained against the chest wall from the IMF cranially over a span of 4 cm. **b** Path of the cutaneous branches of T4 and T5 nerves as they exit the chest wall. Credit for illustrations: Flora Varghese (co-author), 2018.

Although the definition has not been standardized, PMPS is thought to be a type of neuropathic pain or a complex chronic pain state, which is typically associated with nerve fiber injury^14^. Our data suggest that at least some women with this condition likely have a formation of neuromas that arise as a consequence of nerve injury, where the T4 and T5 cutaneous intercostal nerve branches enter the breast tissue during procedures such as mastectomy and reduction mastopexy. The areas of maximal point tenderness correlate with the anatomic location of these nerves as they exit the chest wall to innervate the breast (Fig. 2). For those women who form painful neuromas, this can happen either very quickly postoperatively or in a delayed fashion. We evaluated the efficacy of a treatment modality in women with post-operative pain likely related to neuroma formation.

Post-mastectomy pain syndrome occurs commonly after breast surgery and can be debilitating for the patient. The presentation often includes sensitivity to light touch and pain that radiates across the chest wall or to the shoulder. Chronic pain, sensitivity, and burning sensations can interfere with daily activities such as driving, the ability to wear a seatbelt, as well as wearing a brassiere or any clothing that comes in contact with the trigger point. This constellation of symptoms is consistent with neuropathic pain, which is classically associated with an abnormal response to non-painful tactile stimulus (allodynia) and exaggerated or heightened response to a painful stimulus (hyperalgesia)^6^. The constellation of symptoms correlates with the anatomy of the nerves and their distribution, which is what originally alerted us to both the origin of the problem and the possible solution. Following a successful trigger point injection, the pain at the site is relieved, as well as the radiation of pain and sensitivity to light touch across the chest wall or shoulder (see Fig. 2 for nerve distribution).

Patients often report that their symptoms are ignored, not taken seriously, or blamed on mental health issues^15^. It is important to educate women and surgeons that injury to the T4 and T5 nerves and the development of chronic pain, presumably due to neuromas resulting from breast surgery, can be prevented and treated. Our data suggest that for many women if a trigger point is identified, this significant problem can easily be solved with a simple injection or series of injections performed in an outpatient clinic setting. A long lapse of time from the surgical procedure where the pain originated was, surprisingly, not a predictor that the intervention will be ineffective. Although major complications associated with surgery were more predictive of failure to achieve long-term relief, this should not dissuade the attempt to relieve pain with a simple trigger point injection. We demonstrated the vast majority of these trigger points can be relieved with a simple intervention conducted by surgeons during an office visit. Some patients require repeated injections to eventually achieve relief. For women who achieved initial resolution of their pain, more moderate pain can occasionally recur months or years later but was then typically amenable to a repeat injection.

Unfortunately, a small percentage of our patients did not derive long-term pain relief with trigger point injections. There are theories to explain why some neuromas are not relieved by TPI. Once the nerve is injured in surgery, it undergoes demyelination, which initially affects ascending pain fibers at the site of the nerve injury. Neuropathic pain is a chronic and dynamic phenomenon that displays plasticity on the gross, cellular, and molecular levels over time within the spinal cord. This leads to changes in other parts of the afferent sensory pathway, that ultimately cause a decrease in descending pain-inhibitory signals^16,17^. Without this critical inhibitory pathway, ascending pain signals are amplified, often in the absence of a stimulus, and may even continue after the inciting factor has been eliminated^16–18^.

Although the mechanisms of intractable neuropathic pain are not completely understood, clinicians and scientists have identified molecular pathways in animal models that may be contributory. Murine models with induced partial sciatic nerve injury have revealed an upregulation in the expression and release of lysophosphatidic acid. This contributes to crosstalk between noxious and tactile nerve fibers and can encourage sprouting of injured nerve endings after demyelination at the injury site^16^.

Prevention of post-mastectomy pain should also be emphasized. Surgeons operating near the course of the T4 and T5 intercostal nerves should take care to identify, clip, and cut nerves as needed rather than merely cauterizing them. This helps prevent so-called sprouting and crosstalk between damaged nerve endings and intact nerves in the surrounding area^16^. In the areas of the T4 and T5 intercostal nerve entering the chest wall, special care should be taken to search for vessels that have an accompanying nerve branch and separate the vessel from the nerve prior to cauterizing the vessel. These branches of the intercostal arteries are most likely to be found at angles to the vertical of 6 and 9 o’clock on the right, and 6 and 3 o’clock on the left.

Perineural trigger point injection with a combination of bupivacaine and dexamethasone is a safe, simple, and effective treatment option for post-mastectomy pain syndrome. Surgeons performing breast operations should inquire about the presence of symptoms consistent with PMPS in the immediate post-operative period and understand the importance of early intervention in treating neuropathic pain. This technique should be added to the armamentarium of all surgeons who perform breast surgery.

## Methods

### Patient selection

The study population included women who underwent breast surgery and developed persistent PMPS as identified by the presence of trigger points along the inframammary fold. All patients were examined and treated by a breast surgeon at the UCSF Breast Care Center, and informed consent was obtained. The study was approved by the Institutional Review Board of the University of California San Francisco.

### Intervention

Each trigger point identified on a physical exam was used to direct injection, consisting of a 2 mL mixture of equal parts 0.5% bupivacaine with 4 mg/mL dexamethasone. Standard alcohol or chlorhexidine prep was used to clean the skin prior to injection. The target of the injection is the soft tissue just above the chest wall at the site of the trigger point, and the mixture was massaged in the region of the trigger point for 1–2 min. This is the same dose combination used by the UCSF anesthesia department pain management team for similar pain etiologies and is a standard of care intervention. The location of the trigger point tenderness was consistently observed along the inframammary fold in the region of 3 and/or 6 o’clock (left breast) or 9 and/or 6 o’clock (right breast) as shown in Fig. 2. The trigger point was used to mark the skin for the site of the injection. The injection was carried out using a 30-gauge needle with the intent to deposit the mixture in the perineural space where T4 or T5 cutaneous nerve branches exit the chest wall and enter the breast and subcutaneous tissue.

### Assessment

The number of trigger points and the injection locations were documented prospectively and verified by chart review. Every patient was assessed with regard to the effectiveness of the TPI. This was obtained by physical examination immediately (1–3 min) after the injection, followed by a long-term assessment with a telephone interview conducted at least 3 months after the intervention. Patients who had persistent or recurrent pain were offered a follow-up appointment for repeat injections. Patients whose pain recurred after a delay of several months returned for repeat injections and were re-treated as needed. Ethics approval was waived by the Institutional Review Board as this was a quality improvement project and required telephone interview follow-up by physicians who did not conduct the procedure to assess the long-term outcome of the intervention. Permission was granted to publish the results. The trigger point injection was a clinical care quality improvement initiative using a standard pain management intervention. Patients received the intervention as part of the standard of care, and the study was to assess outcome and as such there was no requirement for written informed consent.

### Telephone interview

Telephone interviews were conducted between January 2016 and April 2018. Two of the patients were deceased at the time of the phone survey and were therefore excluded from the study. The following questions were asked:

1. Has your pain resolved? (Yes/No)
  - If Yes, for how long (time in months)?
  - If No, did you have temporary relief (Yes/No)?
    i. If Yes, how long (time in months)?
    ii. If Yes, are you requiring ongoing oral analgesia (Yes/No)?
      1. If Yes, what are you taking (descriptive)?
    iii. If No, what are you taking (descriptive)?
    iv. If No, would you consider a repeat injection (Yes/No)?

Satisfactory resolution of pain was our primary endpoint. The pain was determined to be adequately resolved if the patient reported any of the following: subjective features of long-term relief of pain (≥3 months), significant improvement in their quality of life, or self-reported discontinuation of adjunct therapy specifically targeted for the treatment of their pain. Such therapies included NSAIDs, gabapentin, opioid analgesics, or other adjuvant analgesics. The primary endpoint was assessed at the time of the telephone interview.

### Descriptive statistics

Descriptive statistics were retrieved from the electronic medical record (Apex Systems EHR), the Department of Surgery, University of California San Francisco. These included age, number and type of breast and axillary surgical interventions, months with pain prior to intervention, history of surgical complications or infections, history of radiation therapy at the affected side, number of injections required, location of trigger points, and dates of injection. Specifically, the surgical procedures consisted of mastectomy, partial mastectomy with or without reduction mammoplasty, axillary sentinel lymph node biopsy, and axillary lymph node dissection. Surgical complications were categorized as major or minor, which was derived from the Clavien-Dindo (CD) complications severity grading system, where minor consisted of CD grade I, and major CD grade II or higher^12^. The intent of this study was to determine the efficacy of the injection technique for long-term relief of symptoms and the number of injections required for treatment success. Univariate and bivariate analyses were conducted using Stata 12 (College Station, TX).

### Reporting summary

Further information on research design is available in the Nature Research Reporting Summary linked to this article.

## Supplementary information

Reporting Summary

## Data Availability

The data generated and analyzed during this study are available from the figshare repository and described in the following data record: 10.6084/m9.figshare.14885595^13^. Additional information on dates of injections can be made available upon request to the corresponding author.
